# Midshaft tibial osteotomy and bone transport for tibiocalcaneal arthrodesis

**DOI:** 10.3389/fsurg.2026.1758515

**Published:** 2026-02-26

**Authors:** Sunwen Pan, Bo Wang, Zeyu Zhao, Xiaokang Gong, Yueliang Zhu, Zhen Shi

**Affiliations:** 1 Department of Orthopedics, The Second Affiliated Hospital, Zhejiang University School of Medicine, Hangzhou, China; 2 Key Laboratory of Forensic Medicine, Department of Forensic Medicine, School of Basic Medicine, Xinjiang Medical University, Urumqi, China; 3 Department of Orthopedics, 920th Hospital of Joint Logistics Support Force of PLA, Kunming, China; 4 Department of Orthopedics, Hang Zhou Tianmushan Hospital, Hangzhou, China; 5 Department of Burn and Plastic Surgery, Tangdu Hospital, Fourth Military Medical University, Xi'an, China

**Keywords:** ankle arthrodesis, hindfoot deformities, Ilizarov distraction osteogenesis, minimally invasive osteotomy, TC arthrodesis

## Abstract

**Background:**

Talar necrosis and infection present a significant challenge, frequently requiring talectomy, which transforms the tibiotalar joint into a tibiocalcaneal articulation. Key management uncertainties persist regarding the necessity of formal arthrodesis vs. the adequacy of a stable fibrous union, and the optimal method for concomitant limb length restoration. This study evaluates a technique combining minimally invasive midshaft tibial osteotomy with Ilizarov distraction osteogenesis to address tibiocalcaneal reconstruction and limb lengthening, seeking to inform these clinical decisions.

**Methods:**

Twelve patients who underwent TC arthrodesis at the 920th Hospital of the PLA between January 2014 and July 2019 were included. The cohort consisted of 7 cases of talar infection following open fractures and 5 cases of tuberculous talar infection, all with partial or complete necrosis. Outcomes assessed included bone elongation, fusion rates, AOFAS ankle-hindfoot scores, and postoperative complications.

**Results:**

All patients were followed up for 1.5 to 4.5 years. The external fixation frame was maintained for an average of (3.04 ± 0.32) months. Bone transport ranged from 4.3 to 7.0 cm, with a mean of (5.88 ± 1.00) cm. Tibial-calcaneal fusion was achieved in 7 cases, while 5 cases exhibited pseudarthrosis; however, their daily activities were unaffected, and pain levels were mild. The average AOFAS score was (75.92 ± 3.73) postoperatively (*p* < 0.0001), indicating a marked enhancement in functional outcomes with no recurrent infections or postoperative complications.

**Conclusion:**

This study highlights the role of minimally invasive midshaft tibial osteotomy in optimizing TC arthrodesis outcomes, achieving functional improvements even in cases of pseudarthrosis. Future research should focus on management protocols for pseudarthrosis to further enhance TC arthrodesis effectiveness.

## 1 Background

The management of infectious talar defects presents a persistent clinical challenge. Radical debridement for chronic osteomyelitis following open talar injuries, which demonstrates a reported incidence of 41.2%, inevitably creates substantial bone defects that complicate subsequent reconstruction (1, 2). In the context of specific infections, such as tuberculosis affecting the muscles and bones, the ankle and foot are less frequently affected, accounting for a mere 1% of all tuberculosis infections. Although clinically uncommon, such occurrences are not considered rare (3). Irrespective of the specific type of infection, resultant wounds and sinus tracts manifest prolonged recalcitrance. Traditionally, the standard approach has encompassed meticulous debridement, infection control, skin flap coverage, and subsequent iliac or tibial bone grafting with ankle fusion (4). However, this course of action is characterized by protracted treatment periods, multiple surgical interventions, and a propensity for eventual limb amputation (5).

While a wealth of clinical literature exists pertaining to tibiotalocalcaneal (TTC) arthrodesis, there remains a relative paucity of reports detailing tibiocalcaneal (TC) arthrodesis (6). Tibiotalocalcaneal arthrodesis remains a viable option in cases of aseptic necrosis of the talus (7), However, in cases of tibiofibular infection, osteomyelitis, whether due to tuberculosis or bacterial infection, or the occurrence of talus necrosis, TC arthrodesis is the only option (8). There are various methods for TC arthrodesis, with autogenous cancellous bone grafting being commonly used. If not, limb shortening may occur after fusion (9). Alternatively, membrane-induced osteogenesis techniques can be used (10). Both of these methods require bone grafting from the donor site. On the other hand, the Ilizarov method involves slow distraction osteogenesis without the need for bone grafting, which also carries a low risk of reinfection (11).

In traditional techniques, minimally invasive osteotomy of the proximal tibia and the use of external fixators are cumbersome, inconvenient for daily activities. In conventional techniques, the implementation of minimally invasive proximal tibial osteotomy and the application of external fixation pose notable challenges in terms of practicality and aesthetic considerations (12). This is primarily attributed to the presence of two rings situated on the tibial plateau accompanied by multiple K-wires traversing the affected area. To ensure stability, these metallic wires must be carefully angled as they pass through the knee, often intersecting with the pes anserinus, a tendon structure. Consequently, during knee flexion and extension, the wires may impinge upon tendons and breach the integument, resulting in discomfort and pain (13–17). If minimally invasive osteotomy is performed in the middle segment of the tibia and a smaller bone transport configuration is installed, along with minimally invasive osteotomy, it may offer a safe and comfortable solution to address the bone and soft tissue defects following tibiofibular resection, while preserving the limb and avoiding amputation. Therefore, this retrospective study assessed a tibiocalcaneal arthrodesis technique based on a midshaft, minimally invasive tibial osteotomy in a series of 12 patients. The procedure aimed to achieve ankle reconstruction via single-stage Ilizarov bone transport without the need for bone grafting. What's more, our investigation not only evaluated the fusion rate but also reports a key finding: stable functional outcomes can be achieved independently of complete bony union in such reconstructions.

## 2 Materials and methods

### 2.1 General information

This study included patients who underwent tibiocalcaneal arthrodesis at the 920th Hospital of Joint Logistics Support Force of PLA between January 2014 and July 2019. The cohort comprised 7 cases with talar infection secondary to open fractures and 5 cases with tuberculous talar infection, all presenting partial or complete talar necrosis. The study was approved by the hospital ethics committee, and all participants provided informed consent (Table 1).

**Table 1:** Patient demographics and clinical characteristics.

Patient characteristics	Number of patients (*n* = 12)
Sex	
Male	9
Female	3
Age	
>40	6
<40	6
Etiology	
Road accident injury	3
Crush injury	4
Tuberculosis	5
Injured part	
Right foot	6
Left foot	6
Both feet	0
Diagnosis	
Talus infection	7
Talus tuberculosis	5

### 2.2 Preoperative preparation

Before surgery, comprehensive medical histories were collected, and routine preoperative examinations were conducted. Wound secretions were cultured for bacterial identification and drug sensitivity testing. Patients with ankle joint tuberculosis were evaluated to rule out active pulmonary tuberculosis. The American Orthopedic Foot and Ankle Society (AOFAS) scoring system was used to assess ankle joint function, considering pain, alignment, and foot function. Patient satisfaction levels were categorized as very satisfied, satisfied, average, or dissatisfied.

The extent of talar bone damage was determined through anteroposterior and lateral x-rays of the ankle joint, as well as magnetic resonance imaging (MRI). Based on the radiographic measurements, the extent of talar bone loss was quantified to determine the required bone transport distance. The osteotomy plane was planned in the mid-diaphyseal region of the tibia. For patients diagnosed with or suspected of ankle joint tuberculosis, a course of oral anti-tuberculosis drugs (isoniazid, rifampicin, ethambutol, pyrazinamide) was administered for 2–3 weeks. Surgery was not recommended if symptoms did not improve or if tuberculosis drug treatment proved ineffective.

### 2.3 Surgical methods

#### 2.3.1 Debridement

Under general anesthesia with endotracheal intubation, both lower limbs were prepared following a standard aseptic protocol involving sequential scrubbing with hydrogen peroxide, povidone-iodine, and normal saline. The ankle joint was then positioned neutrally, and the superficial peroneal nerve and dorsalis pedis artery were marked for reference. A meticulous exploration of the ankle joint was performed through the existing open wound or via an anteromedial incision between the anterior tibial tendon and the medial malleolus.

The entire talus, along with all infected subchondral bone surfaces of the distal tibia, was radically excised. The talus was removed either *en bloc* or piecemeal using rongeurs until healthy, bleeding bone margins were reached. All necrotic and infected soft tissues were thoroughly debrided.

Following complete debridement, the wound was copiously irrigated with a minimum of 3,000 mL of normal saline. All surgical instruments, gloves, and drapes were then changed. The surgical field was reprepped and draped to establish a new sterile environment before proceeding to the next stage of reconstruction.

#### 2.3.2 Ilizarov bone transport

##### 2.3.2.1 Frame application and limb alignment

An Ilizarov external fixator was applied. Proximally, two fixation rings were mounted on the tibia, with the most proximal ring placed distal to the pes anserinus to minimize irritation to this tendon complex. Distally, a single ring was fixed to the tibial segment to be transported, and a separate foot ring was secured to the calcaneus. Each ring was stabilized with either two transosseous wires or a combination of one wire and one half-pin. Critical technical note: The wires for the distal tibial transport ring engaged the tibia only, avoiding the fibula to allow unimpeded segment transport. In contrast, wires for the proximal tibial rings could transfix both the tibia and fibula to enhance stability.

Before final tightening, the foot was positioned to achieve the preoperatively planned tibiocalcaneal alignment: a neutral sagittal position (approximately 90° of ankle flexion) with 5° of external rotation, meticulously matching the posterior heel prominence to the contralateral limb. This alignment ensured that the final tibiocalcaneal relationship would optimally mimic the contralateral side for postoperative function and footwear compatibility.

##### 2.3.2.2 Midshaft tibial osteotomy

**Figure 1:**
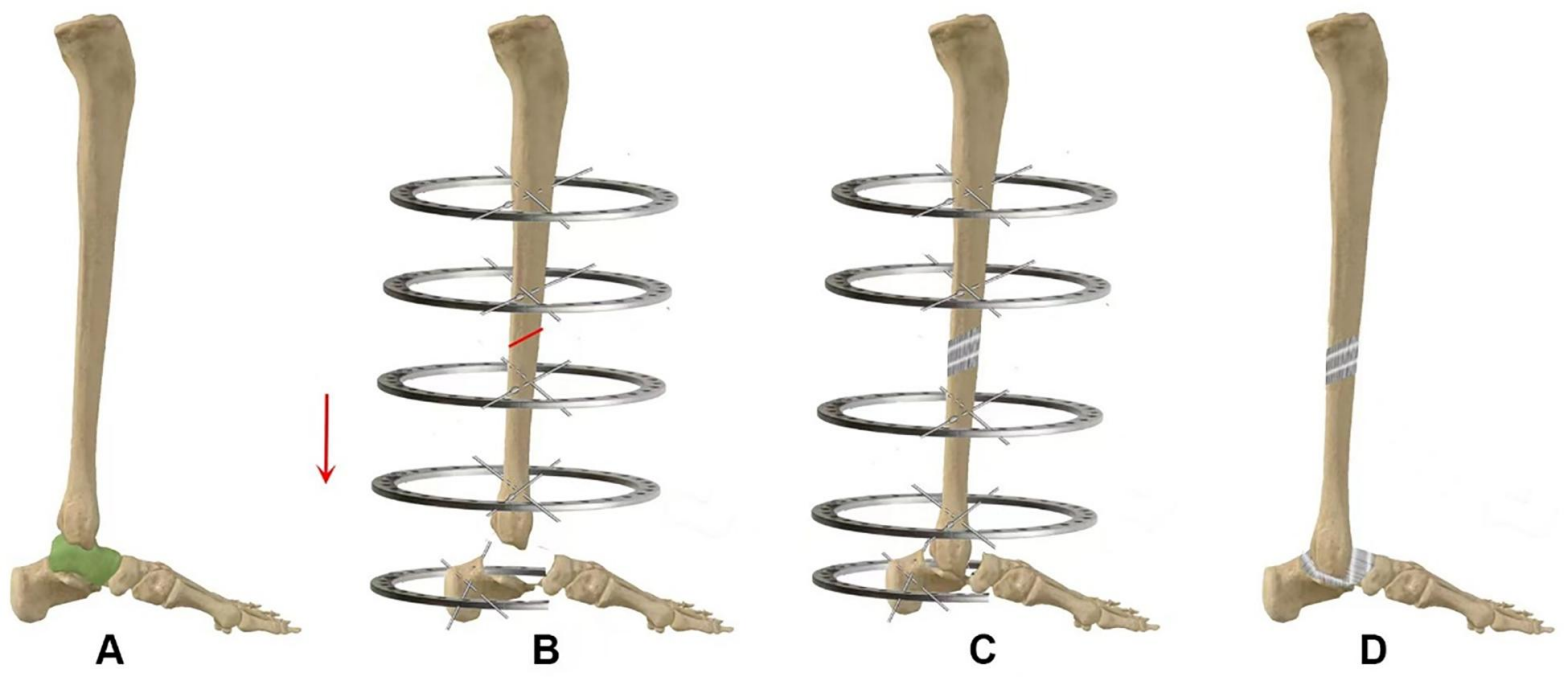
Surgical technique schematic diagram. **(A)** Astragalectomy. **(B)** Postoperative talus removal and minimally invasive midshaft tibial osteotomy. **(C)** Distraction osteogenesis of the middle tibia. **(D)** Fusion of the distal tibia with the calcaneus and removal of the Ilizarov external fixation frame after fusion.

A minimally invasive osteotomy was then performed at the preoperatively planned mid-diaphyseal site via two 1-cm anterior skin incisions. Using sharp osteotomes, the anterior, interosseous, and lateral cortices were disrupted through the lateral incision, and the medial cortex through the medial incision. To avoid thermal necrosis, powered drills were not used. The osteotomy was completed using the osteotomes and a mallet; a controlled final fracture of any remaining bony bridges was achieved by manually applying torsional force to the osteotome handle. After completing the osteotomy and releasing the connecting rods between the proximal and distal ring blocks, the tibia was confirmed to be fully separated. Final alignment of the transport segment was achieved by adjusting the distal ring. The schematic diagram of the surgical technique is illustrated in Figure 1.

### 2.4 Postoperative management

Standard postoperative care was implemented, focusing on infection control, edema reduction, and pain management. Pin-site hygiene was maintained using a Pikus needle to clean any serosanguineous discharge promptly with saline or povidone-iodine solution to prevent infection. Throughout the external fixation period, pin sites were disinfected and dressed in sterile gauze weekly.

Distraction was initiated after a one-week latency period at a rate of 0.6 to 1 mm per day, divided into three to four adjustments. The process continued until the transported tibial segment achieved close apposition with the calcaneus, confirmed by serial radiographs. Adjustments were temporarily paused for 1–2 days if the patient reported significant pain during elongation.

Upon completion of transport, the frame was maintained in a static, neutralization mode for a minimum of three additional months to allow for consolidation at both the docking site and the midshaft regeneration. The total frame time therefore comprised the transport period (variable, dependent on defect length) plus a minimum 3-month consolidation period. Regular radiographic examinations were conducted monthly to assess bone healing. The decision for frame removal was based on evidence of sufficient bone consolidation at the osteotomy site, judged by bridging callus on at least three cortices on anteroposterior and lateral views. Complete radiographic mineralization was not an absolute prerequisite for removal if clinical and radiographic stability were confirmed.

### 2.5 Perioperative antimicrobial and anti-tuberculosis therapy

Regarding antimicrobial management, a standardized perioperative protocol was implemented. For post-traumatic cases, empirical intravenous antibiotic prophylaxis with a second-generation cephalosporin (e.g., cefazolin 2 g) was administered within 1 h prior to incision. This was continued postoperatively for a total duration of 7 days (18, 19). In all cases of open fracture, deep tissue specimens were obtained intraoperatively for microbiological culture and sensitivity testing, and the empirical regimen was subsequently adjusted based on these results to ensure targeted therapy. For patients with talar tuberculosis, a complete, guideline-adherent anti-tuberculosis chemotherapy regimen (2-month intensive phase with four drugs followed by a 12-month continuation phase with two drugs) was administered under specialist supervision, spanning the pre- and postoperative periods (3, 20, 29).

### 2.6 Statistical methods

Continuous variables, including bone elongation length, external fixation duration, follow-up duration, and AOFAS scores, are presented as mean ± standard deviation. Preoperative and postoperative AOFAS scores were compared using paired t-tests. Statistical significance was defined as a p-value < 0.05. All statistical analyses were performed using SPSS software (version 26.0, IBM Corp., Armonk, NY, USA).

## 3 Results

All 12 patients included in the study were successfully followed up, with a duration ranging from 1.5 to 4.5 years. The bone transport phase (from osteotomy to docking) required a mean duration of 3.04 ± 0.32 months (range, 2.5 to 3.5 months) of external fixation. The achieved bone lengthening ranged from 4.3 to 7 cm, with an average of 5.88 ± 1.00 cm. In terms of tibiocalcaneal joint conditions, 7 cases (58.3%) achieved complete fusion, while 5 cases (41.7%) exhibited pseudarthrosis (incomplete fusion). Patients with pseudarthrosis had an AOFAS score of 77.43 ± 3.56, while those with complete fusion scored 73.80 ± 3.11, indicating significant improvements in both groups (*p* < 0.0001), and patients' daily activities were not affected, with mild and acceptable pain levels, negating the need for a second fusion surgery. Overall, the AOFAS ankle-hindfoot score was determined to be (75.92 ± 3.73) points postoperatively (*p* < 0.0001), with 9 cases considered above good, 3 cases rated as fair, and no cases classified as poor. The overall excellent/good rate was calculated to be 75%. None of the 12 patients experienced infection recurrence during the follow-up period. All surgical wounds achieved clinical healing, and there were no instances of post-removal complications such as fractures or traumatic osteomyelitis. The presented cases exemplify the typical outcomes observed in this study (Table 2).

**Table 2:** Individual surgical outcomes and comparative functional scores.

Patient number (*n* = 12)	Lengthening achieved (cm)	Transport time (months)	Fusion status	Pre-op AOFAS	Final AOFAS
Complete fusion (*n* = 7)					
1	6.3	3.5	Complete	25	77
2	6.9	3	Complete	35	84
3	4.6	3.4	Complete	34	76
4	4.8	2.5	Complete	29	73
5	7	2.7	Complete	30	76
6	6.6	3.4	Complete	36	80
7	7	3.1	Complete	42	76
Group summary (Mean ± SD)				33.00 ± 5.54	77.43 ± 3.56
*P* value (pre- vs. post-op AOFAS)					<0.0001
Pseudarthrosis (Incomplete fusion) (*n* = 5)					
8	6.2	2.6	Pseudarthrosis	27	77
9	5.2	3	Pseudarthrosis	35	70
10	6.5	3	Pseudarthrosis	33	76
11	5.2	3.2	Pseudarthrosis	42	75
12	4.3	3.1	Pseudarthrosis	30	71
Group summary (Mean ± SD)				33.40 ± 5.67	73.80 ± 3.11
*P* value (pre- vs. post-op AOFAS)					<0.0001
Overall cohort Summary (*n* = 12, Mean ± SD)	5.88 ± 1.00	3.04 ± 0.32		33.17 ± 5.34	75.92 ± 3.73
*P* value (pre- vs. post-op AOFAS)					<0.0001

### 3.1 Typical cases presentation

**Figure 2:**
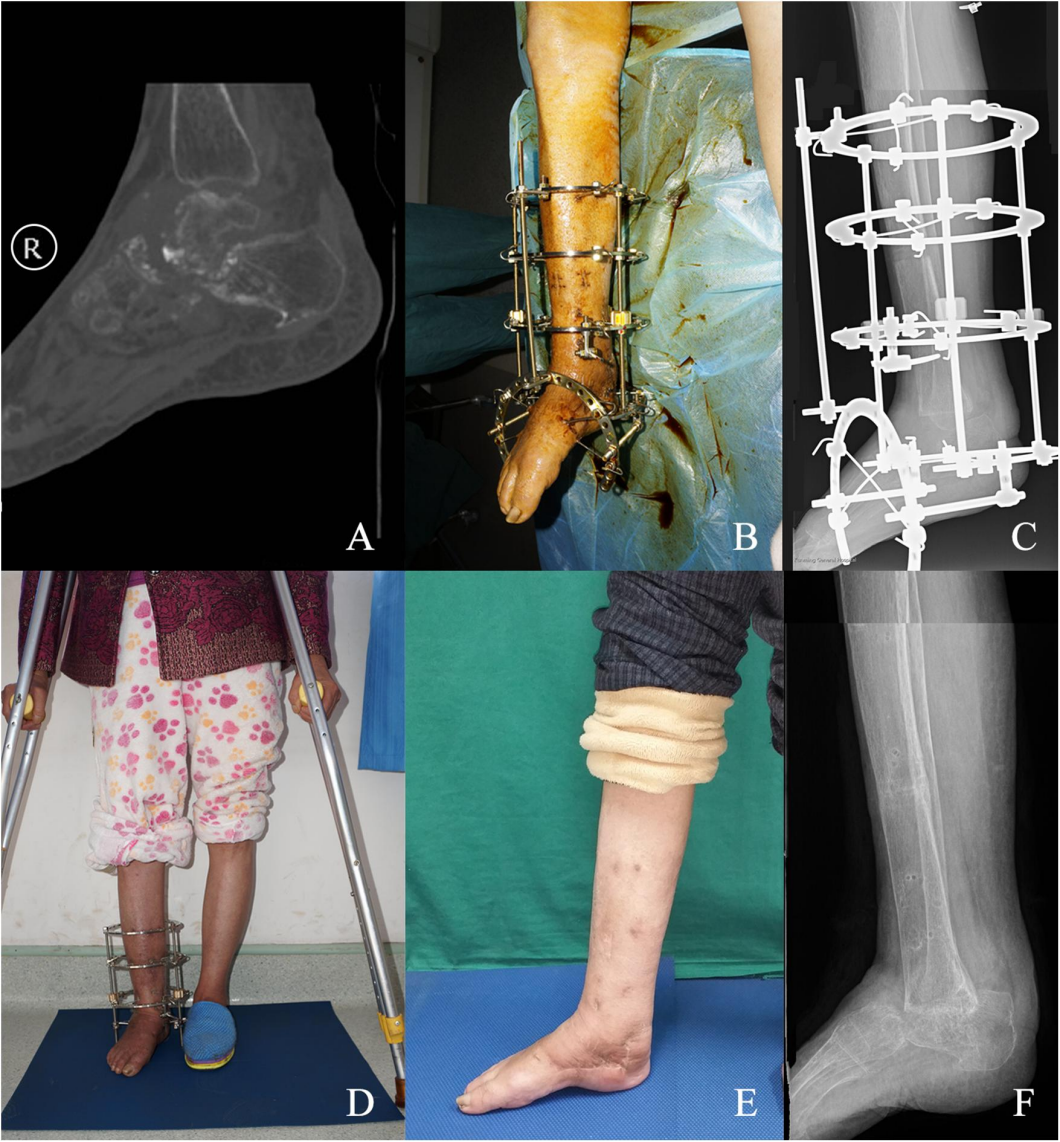
Female patient, aged 58 years: **(A)** preoperative ankle joint plain CT imaging revealed manifestations of ankle joint tuberculosis and talus necrosis, with the absence of active pulmonary tuberculosis. The preoperative AOFAS score stood at 35 points. **(B)** Subsequent to a 3-week course of anti-tuberculosis therapy, the patient underwent talus excision surgery, coupled with partial resection of the distal tibia until achieving normal, uninfected bone integrity. Following meticulous wound debridement, a minimally invasive midshaft tibial osteotomy and tibiocalcaneal fusion procedure were performed. **(C)** Postoperatively, radiographic assessment of the lower limb unveiled a mid-lower segment tibial transection, with the application of an Ilizarov external fixator in a state of bone traction. **(D)** Postoperative ambulation was initiated with the aid of crutches, alongside participation in structured rehabilitation programs, and a continuation of anti-tuberculosis treatment for a duration of six months. A subsequent three-month follow-up x-ray evaluation revealed favorable tibio-calcaneal fusion and commendable bone mineralization in the midshaft of the tibia, prompting the removal of the external fixator. **(E)** A comprehensive two-year follow-up examination demonstrated the patient's report of enhanced lower limb mobility and sensory perception, accompanied by an elevated AOFAS score of 84 points.

Case 1 (Figure 2): A 58-year-old female with tuberculous talar necrosis underwent talectomy and midshaft tibial osteotomy with 6.9 cm Ilizarov bone transport over 3 months, achieving solid tibiocalcaneal fusion and AOFAS score improvement from 35 to 84 at 2-year follow-up.

**Figure 3:**
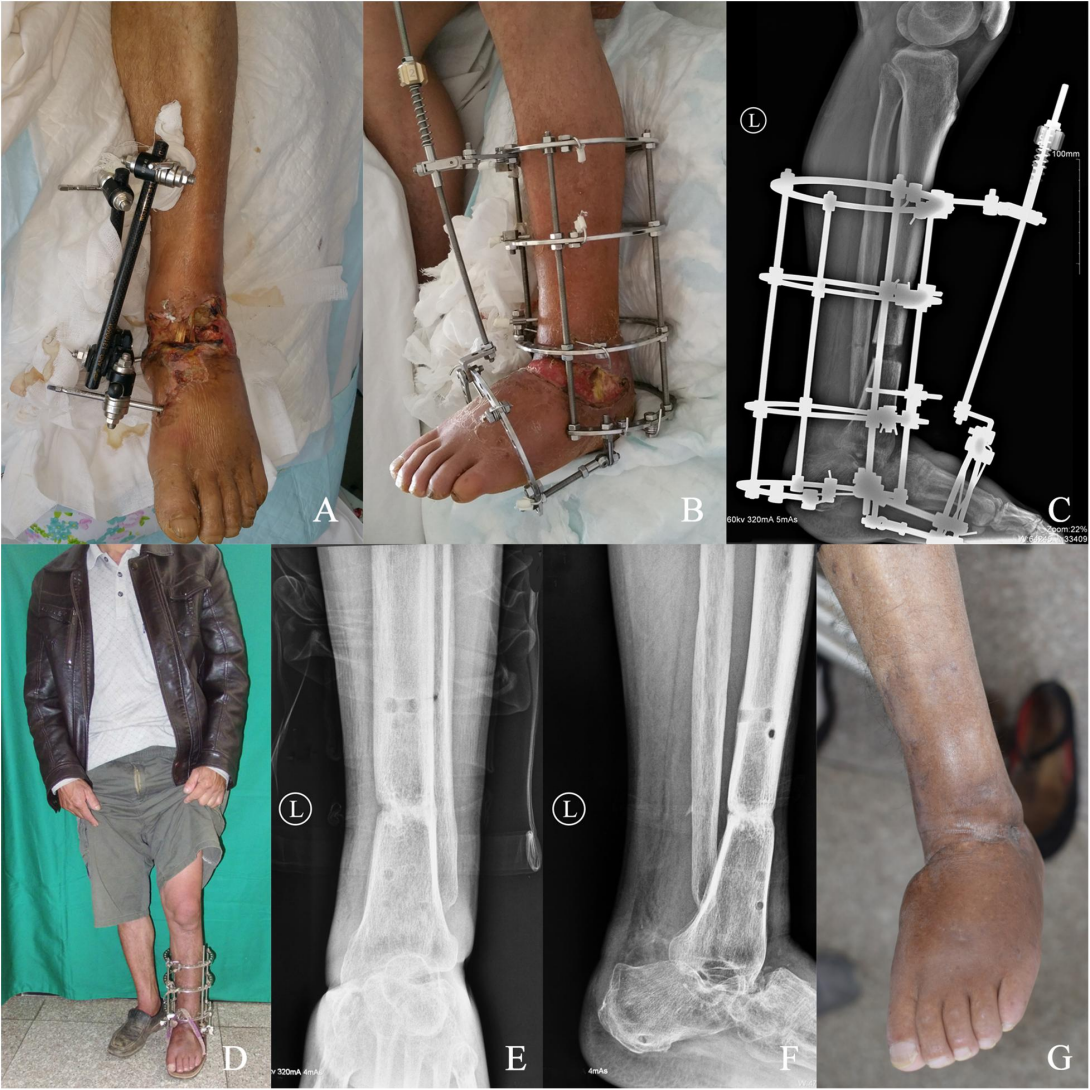
Male patient, aged 45 years: **(A)** preoperatively, diagnostic findings indicated the presence of infectious necrosis involving the talus, in conjunction with soft tissue infection, resulting in an initial AOFAS score of 27 points. **(B)** Following the exclusion of contraindications for surgical intervention, the patient underwent talus excision surgery and concomitant debridement of the affected skin soft tissues. Subsequent to meticulous wound care, a minimally invasive midshaft tibial osteotomy and tibio-calcaneal fusion procedure were meticulously executed. **(C)** Postoperative radiographic scrutiny showcased a mid-lower segment tibial transection, with the utilization of an Ilizarov external fixator in a state of bone traction. **(D)** Postoperative mobilization was initiated with the aid of crutches, complemented by a structured regimen of rehabilitation exercises. **(E** and **F)** A follow-up examination at two and a half months postoperatively revealed suboptimal tibio-calcaneal fusion, culminating in the formation of pseudoarthrosis. Noteworthy bone mineralization in the midshaft of the tibia necessitated the removal of the external fixator. **(G)** An extensive 1.8-year postoperative follow-up evaluation revealed the patient's satisfactory lower limb mobility and sensory function, with the pseudoarthrosis exhibiting no discernible impact on routine ambulation or physical activities, yielding an AOFAS score of 77 points.

Case 2 (Figure 3): A 45-year-old male with post-traumatic infectious talar necrosis underwent the same procedure with 6.2 cm bone transport over 2.6 months; despite pseudarthrosis at the docking site, AOFAS score improved from 27 to 77 at 1.8-year follow-up with no functional impairment.

## 4 Discussion

Arthrodesis is a commonly employed method for end-stage pathologies and neuropathic changes affecting the ankle joint and hindfoot. Although it entails sacrificing joint mobility, it allows for the attainment of a plantigrade foot, offering pain relief and stability in the hindfoot region, thus meeting the functional needs of daily activities (21). It is worth noting that TC arthrodesis, or pseudo-TC joint, is rarely mentioned in medical literature. Consequently, there is a paucity of data regarding the effects of tibiocalcaneal (TC) arthrodesis on foot-ankle biomechanics, changes in patient satisfaction, and long-term outcomes. The present case series represents an effort by the authors to contribute useful clinical experiences and insights to this relatively underexplored area of research.

For the treatment of bone defects, the Ilizarov distraction osteogenesis technique is primarily utilized. This technique applies the tension stress law, which states that continuous and gradual traction stimulation can promote tissue growth (22). Thus, the defected talus could be replaced with a distally lengthened tibia to join the proximal calcaneus to form a new “joint” or just connect the calcaneus to recover the continuality of the limb. With the help of the regeneration ability of Ilizarov technique, the surgeons could do a radical debridement, completely removing infected bones and tissues (23); The Masquelet technique is also frequently employed for repairing infected bone defects and involves two stages: (i) the use of an antibiotic bone cement spacer after thorough debridement (T1), and (ii) the transplantation of an induced membrane and cancellous bone graft 6–8 weeks after spacer removal to promote bone defect repair(T2) (24). However, this technique requires a safe soft tissue coverage, which means additional flap transfer, if there is an open wound or infected wound. Besides, some patients would refuse the bone graft taken from other donor sites like an anterior or posterior superior iliac spine (24, 25). In comparison, the Ilizarov technique allows for early weight-bearing and can be completed in a single stage.

Simultaneously, dysplasia of distraction osteogenesis of the tibia bone transport may occur if there is excessive involvement of the osteotomy site. As traditional fretsaw or pendulum saw osteotomies could cause significant damage to the bone graft site and result in slower bone healing, we abandoned this technique many years ago. Some scholars have described the utilization of percutaneous perforated or stamp-style osteotomes to perform meticulous tibial osteotomies, with the aim of preserving the periosteal attachments and facilitating more favorable outcomes with subsequent distraction osteogenesis (26, 27). However, this approach has been associated with an undesirable consequence - thermal necrosis of the bone marrow due to the heat generated by the powered drill bits (28).

In an effort to completely avoid this thermal injury, we have progressively modified their surgical technique, entirely abandoning the use of powered drill bits. Instead, they now employ 7–10 mm wide osteotomes and a mallet to carefully chisel through the anterior, medial, posteromedial, and lateral columns of the tibial diaphysis, performing a “cold” osteotomy using solely manual instrumentation. Access to the tibia is typically achieved through a single anterior incision, with an occasional posteromedial incision to transect the posterior cortex. After releasing the connecting rings, the partially osteotomized tibial diaphysis can then be manually fractured, taking care to preserve any remaining cortical bone bridges.

Prior to each osteotomy, the surgical team meticulously sharpens the osteotomes to an exceptionally keen edge, to the point where they can directly penetrate the skin without the need for a scalpel incision. This attention to instrument sharpness is of critical importance, given the relatively tenuous blood supply to the tibial diaphysis compared to the metaphyseal regions. By abandoning the use of powered tools and relying solely on manual techniques, the authors have completely avoided the thermal necrosis associated with drill-based osteotomies. This delicate and gentle osteotomy method represents a key technical refinement that the authors believe enhances the success of subsequent distraction osteogenesis for complex tibial defects.

Additionally, a critical technical modification in our protocol was the selection of the osteotomy site. We performed the corticotomy at the mid-diaphysis of the tibia rather than the conventional proximal metaphyseal region. While proximal metaphyseal osteotomies are recognized for their rich blood supply and potentially faster regeneration formation, they necessitate the placement of fixation rings near the tibial plateau. This often requires transfixing wires to traverse the pes anserinus tendon complex, which can cause significant discomfort during knee flexion and extension, adversely affecting patient tolerance and long-term compliance. By shifting the osteotomy to the midshaft, we enabled the placement of the proximal fixation rings distal to the pes anserinus. This strategic positioning minimized irritation around the knee joint, thereby significantly improving patient comfort during ambulation while wearing the frame and enhancing overall adherence to the prolonged treatment regimen. This approach, coupled with the benefits of preserving the nutrient artery and allowing for a more refined frame construct as previously discussed, represents a patient-centered refinement in the technique for complex lower limb reconstruction.

It is worth noting that in 5 cases, complete fusion between the distal tibia and calcaneus was not achieved. The establishment of a fibrous connection between the tibia and calcaneus gives rise to the formation of “pseudarthrosis” at the tibia-calcaneal joint. The patient's AOFAS score was (73.80 ± 3.11). Although this is a limited number of cases and there is no statistically significant difference in AOFAS scores when compared to patients with complete fusion between the tibia and calcaneus (77.43 ± 3.56), the average AOFAS scores improved significantly compared to preoperative scores. There was noticeable improvement in forefoot and hindfoot mobility, gait, and daily activities. The patient's walking and overall activity levels improved. Does this result mean that the arthrodesis of the tibia-calcaneal joint is not mandatory? Further case-controlled studies are needed to determine whether complete fusion is necessary in TC arthrodesis procedures. Due to a lack of data regarding the effects TC arthrodesis on foot-ankle biomechanics, changes in patient satisfaction, and long-term outcomes. The present case series represents an effort by the authors to contribute useful clinical experiences and insights to this relatively underexplored area of research. This study has several limitations that should be acknowledged. First, the sample size is small (*n* = 12), which may affect the statistical power and generalizability of the findings. Second, the retrospective design and lack of a control group preclude causal inferences and direct comparisons with alternative treatments. Future prospective studies with larger cohorts and matched controls are needed to validate our preliminary results and further elucidate the role of midshaft tibial osteotomy in TC arthrodesis.

## 5 Conclusion

This study demonstrates that midshaft tibial osteotomy combined with Ilizarov bone transport effectively addresses the challenge of talar defects resulting from infections through the implementation of tibial-calcaneal fusion. This less commonly utilized approach achieved a mean bone elongation of 5.88 cm, with significant functional improvements observed even in cases of pseudarthrosis. These findings indicate that satisfactory outcomes can be attained despite the absence of complete fusion, underscoring the necessity for further research on the management of pseudarthrosis in TC arthrodesis.

## Data Availability

The raw data supporting the conclusions of this article will be made available by the authors, without undue reservation.
